# Single Nucleotide Polymorphisms of One-Carbon Metabolism and Cancers of the Esophagus, Stomach, and Liver in a Chinese Population

**DOI:** 10.1371/journal.pone.0109235

**Published:** 2014-10-22

**Authors:** Shen-Chih Chang, Po-Yin Chang, Brendan Butler, Binh Y. Goldstein, Lina Mu, Lin Cai, Nai-Chieh Y. You, Aileen Baecker, Shun-Zhang Yu, David Heber, Qing-Yi Lu, Liming Li, Sander Greenland, Zuo-Feng Zhang

**Affiliations:** 1 Department of Epidemiology, University of California Los Angeles Fielding School of Public Health, Los Angeles, CA, United States of America; 2 Department of Social and Preventive Medicine, State University of New York at Buffalo, Buffalo, NY, United States of America; 3 Department of Epidemiology, Fujian Medical University, Fuzhou, Fujian, China; 4 Department of Epidemiology, Fudan University School of Public Health, Shanghai, China; 5 Center for Human Nutrition, University of California Los Angeles David Geffen School of Medicine, Los Angeles, CA, United States of America; 6 Department of Epidemiology, Peking University School of Public Health, Beijing, China; 7 Department of Statistics, University of California Los Angeles College of Letters and Science, Los Angeles, CA, United States of America; University of Arizona, United States of America

## Abstract

One-carbon metabolism (folate metabolism) is considered important in carcinogenesis because of its involvement in DNA synthesis and biological methylation reactions. We investigated the associations of single nucleotide polymorphisms (SNPs) in folate metabolic pathway and the risk of three GI cancers in a population-based case-control study in Taixing City, China, with 218 esophageal cancer cases, 206 stomach cancer cases, 204 liver cancer cases, and 415 healthy population controls. Study participants were interviewed with a standardized questionnaire, and blood samples were collected after the interviews. We genotyped SNPs of the *MTHFR*, *MTR*, *MTRR*, *DNMT1*, and *ALDH2* genes, using PCR-RFLP, SNPlex, or TaqMan assays. To account for multiple comparisons and reduce the chances of false reports, we employed semi-Bayes (SB) shrinkage analysis. After shrinkage and adjusting for potential confounding factors, we found positive associations between *MTHFR* rs1801133 and stomach cancer (any T versus C/C, SB odds-ratio [SBOR]: 1.79, 95% posterior limits: 1.18, 2.71) and liver cancer (SBOR: 1.51, 95% posterior limits: 0.98, 2.32). There was an inverse association between *DNMT1* rs2228612 and esophageal cancer (any G versus A/A, SBOR: 0.60, 95% posterior limits: 0.39, 0.94). In addition, we detected potential heterogeneity across alcohol drinking status for ORs relating *MTRR* rs1801394 to esophageal (posterior homogeneity *P* = 0.005) and stomach cancer (posterior homogeneity *P* = 0.004), and ORs relating *MTR* rs1805087 to liver cancer (posterior homogeneity *P* = 0.021). Among non-alcohol drinkers, the variant allele (allele G) of these two SNPs was inversely associated with the risk of these cancers; while a positive association was observed among ever-alcohol drinkers. Our results suggest that genetic polymorphisms related to one-carbon metabolism may be associated with cancers of the esophagus, stomach, and liver. Heterogeneity across alcohol consumption status of the associations between *MTR*/*MTRR* polymorphisms and these cancers indicates potential interactions between alcohol drinking and one-carbon metabolic pathway.

## Introduction

Upper gastrointestinal (GI) cancers are major causes of morbidity and mortality throughout the world. Based on GLOBOCAN 2012 estimates, stomach, liver, and esophageal cancers are the fifth, sixth, and eighth most common cancers, respectively, with a global incidence of approximately 2,189,829 new cancer cases (15.5% of the total), and 1,868,700 deaths (22.8% of the total) [1]. The majority of these cancer cases (1,694,874 cases, 77.4%) occur in less developed countries. China alone accounts for almost half of all incident GI cancers (1,023,072 cases, 46.7%) [1].

Continued research regarding the involvement of single nucleotide polymorphisms (SNPs) in the etiology of these three upper GI cancers has been fruitful. Of particular interest are the SNPs located within genes involved in folate metabolism [2]–[4]. Folate maintains DNA stability by regulating DNA biosynthesis, DNA repair and DNA methylation [5]. Neoplasms may develop when this pathway is disregulated by the depletion of micronutrients or through the incorporation of polymorphisms [5]. Several enzymes are involved in one-carbon metabolism, including the methylenetetrahydrofolate reductase (MTHFR), methionine synthase (MTR), methionine synthase reductase (MTRR), DNA methyltransferases (DNMTs), and mitochondrial aldehyde dehydrogenase 2 (ALDH2). MTHFR, MTR, and MTRR are involved in DNA synthesis, and the generation of S-adenosylmethionine (SAM)—a universal methyl-donor for methylation reactions. DNMTs catalyze DNA methylation and replicate methylation patterns. ALDH2 is responsible for metabolizing acetaldehyde generated during alcohol metabolism. Alcohol and acetaldehyde can inhibit folate absorption and impair DNA methylation [6].

The role of folate and one-carbon metabolism in upper GI cancers is not fully understood. Animal studies provided some evidence for an effect of low folate levels in oxidative stress, DNA methylation, and hepatocarcinogenesis [7], [8]; while high folate intake can increase global DNA methylation and reduce gastric cancer risk [9], [10]. Epidemiologic studies have suggested that genetic polymorphisms of genes in one-carbon metabolic pathway might modulate the risk of esophageal and gastric cancer [4]. However, published results are inconclusive and limited in terms of the number of genes/polymorphisms being investigated. Possible modification by related micronutrients and known risk factors has seldom been explored. Therefore, considering the importance of one-carbon metabolism in upper GI cancer development, we examined the associations between eight SNPs in genes in one-carbon metabolic pathway and cancers of the esophagus, stomach, and liver in a Chinese population. We also evaluated heterogeneity of the associations across different strata of plasma micronutrients (including folate, vitamin B12, and total homocysteine) and known risk factors for these cancers.

## Materials and Methods

### Ethics statement

This study was exempted by the institutional review board of University of California at Los Angeles (Certified Exempt 02-248).

### Study Design and Population

A detailed description of the study design has been published previously [11], [12]. Briefly, this was a population-based case-control study conducted in Taixing City, Jiangsu Province, China. Eligible cases were newly diagnosed patients with pathologically or clinically confirmed esophageal cancer (between June 1 and December 30, 2000), stomach cancer (between June 1 and December 30, 2000), and liver cancer (between January 1 and June 30, 2000) reported to the Taixing CDC Tumor Registry. Other inclusion criteria including being 20 years of age or older, in stable medical condition as determined by a physician, residency in Taixing for 10 years or more, and willingness to participate. A total of 218 esophageal cancer cases, 206 stomach cancer cases, and 204 liver cancer cases participated, representing 67, 65 and 57%, respectively, of all newly diagnosed cancer patients.

Controls were randomly selected among healthy residents of Taixing City with a 2∶3 frequency matching ratio to the combined case group on 5-year age categories (20–24 to 80–84), sex, and residency (village in rural township or in an urban residential block in central Taixing City). There are 23 townships (rural areas) and one central town (urban area) in Taixing City. Each rural township consists of 10–12 villages, and the central urban area consists of 10–12 residential blocks. Other inclusion criteria were the same as the cases. A total of 464 potential controls were approached, and 415 (89.4%) consented to participate.

### Epidemiologic data collection

All of the recruited cases and controls completed a standard questionnaire administered by trained interviewers. Interviews took place either at the participants' homes, in the hospitals (for cases), or in the county doctor's office (for controls). Cancer cases were usually interviewed within 6 months of diagnoses. The questionnaire collected detailed information on demographic factors, current height and weight, dietary history, tobacco smoking history, alcohol drinking history, tea drinking habits, occupational history, family history of cancers, and physical activities.

### Laboratory assays

Each study participant provided a 5-ml peripheral blood sample after their interviews. DNA was isolated from blood clots, using the phenol-chloroform method. Hepatitis B virus surface antigen (HBsAg), IgG antibodies for hepatitis C virus (HCV), and IgG antibodies for CagA-*Helicobacter pylori* (H. *pylori*) were measured by enzyme-linked immunosorbant assay (ELISA) using kits from the Reagent Company of the Shanghai Hospital for Infectious Diseases (Shanghai, China), the Shanghai Huamei Biological Company (Shanghai, China), and the Reagent Company of the Shanghai Biotechnology Industry Park (Pudong, Shanghai, China), respectively. Plasma aflatoxin B1 (AFB1)-albumin adduct levels were determined by ELISA assay, as previously described [13], using free aflatoxin (Supelco) for the aflatoxin standards. A comparison between free and bound aflatoxin standards revealed a log-linear relationship, allowing us to estimate the absolute values of the samples. Plasma folate and vitamin B12 levels were measured using a competitive radioassay with iodine 125-labeled folate and cobalt 57-labeled vitamin B12 as tracers (Quantaphase II B12/folate radiobinding kit, Bio-Rad, CA). Plasma total homocysteine (tHcy) levels were measured using a commercially available chemiluminescent immunoassay system (IMMULITE 1000 Automated Analyzer, DPC, Los Angeles, CA).

We selected eight SNPs from *MTHFR*, *MTR*, *MTRR*, *DNMT1*, and *ALDH2* genes, based on the following criteria: 1) SNPs which are functional or potentially functional (SNPs located in the coding, 3′-, and 5′-untranslated regions); 2) SNPs previously reported to be associated with upper GI cancers; and 3) SNPs with minor allele frequency of at least 5% in the National Center for Biotechnology Information SNP database. Genotyping was performed using the TaqMan (*MTR* rs1805087, *MTRR* rs1532268/rs1801394, and *ALDH2* rs886205) or the SNPlex (*DNMT1* rs2228612 and *ALDH2* rs2238151) assay, as previously described (Applied Biosystems by Life Technologies, Foster City, CA) [14], or the PCR-RFLP analysis (*MTHFR* rs1801133 and *ALDH2* rs671) modified from previously published methods [15], [16]. Genotyping call rates were over 97% for TaqMan and PCR-RFLP methods, and over 80% for the SNPlex assay. Reproducibility was 98% for the SNPlex assay (3% random duplicate samples) [17], and 100% for the TaqMan assay (10% random duplicate samples).

### Statistical analysis

We used Pearson's chi-square test for Hardy-Weinberg equilibrium (HWE) for the distributions of genotype frequencies of the eight SNPs in the controls only. Testing for HWE among the controls is a commonly used preliminary quality-control method in genetic association studies to identify systematic genotyping errors in unrelated individuals. We analyzed each SNP-cancer association under co-dominant, log-linear, dominant, and recessive genetic models, using unconditional logistic regression models to calculate odds ratios (ORs) and 95% confidence intervals (CIs). Models included age-matched categories, sex, residency (urban/rural), education (illiteracy/primary school/higher than middle school), body mass index (BMI, continuous), smoking pack-years (continuous), alcohol consumption frequency (never/occasionally/often/everyday), *H. pylori* infection (stomach cancer; negative/positive), HBsAg status (liver cancer; negative/positive) and plasma AFB1-albumin adduct levels in quintiles (liver cancer; estimated quintile: <222.7, 222.7–344.2, 344.2–442.6, 442.6–588.5, and >588.5 fmol/mg). To adjust for residual confounding effects from age, we also included the deviation of each person's age from the mean age in each age category [18]. We caution that a number of adjustment variables may be affected by genetic variations, as these variables occur afterward. At best, our estimates are for direct genotype effects, and otherwise may be over-adjusted or confounded by uncontrolled factors that affect both the adjustment variables and the outcomes [19]. Thus, we checked estimates for direct genotype effects against estimates adjusted only for age and sex.

We further conducted stratified analyses to check heterogeneity across strata of micronutrients or modifiable risk factors, including plasma micronutrients (folate, vitamin B12, and tHcy), smoking status, alcohol consumption, H. *pylori* infection (stomach cancer), HBsAg status (liver cancer), and plasma AFB1 levels (liver cancer). We used estimated median levels in controls to dichotomize plasma levels of folate (12.76 nmol/l), vitamin B12 (228.88 pmol/l), tHcy (9.5 µmol/l), and AFB1 (388.95 fmol/mg). We used the dominant genetic model, which assumed that the effect of the variant allele is dominant if the ratio of the ORs comparing variant allele homozygotes to heterozygotes was smaller than that comparing heterozygotes to common allele homozygotes; otherwise we used the recessive genetic model. We assessed heterogeneity across strata using likelihood ratio tests by comparing models with and without product terms.

To reduce the risks of multiple-comparison artefacts and sparse-data bias, we used a semi-Bayes (SB) shrinkage (penalized-likelihood) method to estimate genotype coefficients [20]; the odds-ratio estimates we report are the antilogs of these coefficients. Shrinkage estimation has been recommended extensively as an alternative superior to Bonferroni in the statistical literature for eliminating multiple-testing artefacts in comparative studies [21]–[24]. In shrinkage estimation, instead of changing the alpha level, we regress (‘shrink’) the estimates toward zero to a degree proportional to their estimated variances and inversely proportional to the prior variances v. The prior variance plays a role analogous to the adjusted α-level, in that smaller values correspond to more stringent rejection/detection criteria, with α = 0 and v = 0 being the lower limits of adjustment at which rejection of the null becomes impossible. At the other extreme, no adjustment occurs when using the original value of α or a huge (effectively infinite) value for v.

In our study, we assigned a prior variance of 0.50, and a prior median OR = 1 (no association) which results in a 95% prior probability of falling within the interval 0.25, 4. This pulls the observed associations toward the null to the degree that would result if there had been a previous null experiment observing 4/v = 8 cases total and it had been merged with the current data [20], [25]. When differing stratum-specific SNP effects were allowed, such as in stratified analyses, the prior variance was reduced to 0.25, which corresponds to a variance of 0.50 for the coefficient of the stratum-SNP product (interaction). For each SB posterior estimate, we further provide the directional (one-sided) SB P-values, which equal the posterior probability that the point estimate is on the wrong side of the null under the fitted model and the shrinkage priors [26], [27].

To summarize the associations of the 8 SNPs for each of the three upper GI cancers, we constructed a polygenetic risk score (PRS) [28]. The PRS was calculated as the weighted sum of the risk genotype (under either dominant or recessive model as in the stratified analyses) counts, where the weight for each SNP was determined by the semi-Bayes log OR of its association with each cancer in the fully adjusted model. PRS was only estimated among those with complete genotype data on all of the 8 SNPs, which include 126 esophageal cancer cases, 125 stomach cancer cases, 142 liver cancer cases, and 287 controls. The range (maximum minus minimum) of PRS for each cancer was divided into three equally spaced categories; these ranges were 0.11 to 2.05 for esophageal cancer, 0 to 1.91 for stomach cancer, and 0 to 1.40 for liver cancer. Data analyses were performed using SAS 9.1.3 (SAS Institute, Cary, NC).

## Results

Compared to population controls, cancer cases tended to be smokers, had lower BMI, and lower education levels (Table 1). Esophageal and stomach cancer cases were older than the controls, while liver cancer cases were the youngest. Liver cancer patients had the highest male-to-female ratio of 3.53, and were most likely to consume alcohol; esophageal cancer patients drank more frequently than the other cancer cases and controls in this study. For risk factors specific to each cancer site, we did not observe differing frequency of *H. pylori* infection between stomach cancer patients and controls. Compared with controls, liver cancer patients showed a higher percentage of HBsAg positive (65 vs. 25%), anti-HCV positive (9 vs. 3%), and had higher plasma AFB1-albumin adduct levels (30 vs. 20% in the 5^th^ quintile).

**Table 1:** Distribution for selected demographic and health characteristics comparing esophageal, stomach, and liver cancer cases with controls.

	Controls	Cancer Cases					
		Esophageal Cancer		Stomach Cancer		Liver Cancer	
	(N, %)	(N, %)	One-Sided	(N, %)	One-Sided	(N, %)	One-Sided
	(N = 415)	(N = 218)	*P*-value†	(N = 206)	*P*-value†	(N = 204)	*P*-value†
**Age** *, mean ± SD	57.7±11.8	60.6±9.6	0.001	62.8±9.8	<0.001	53.9±13.0	<0.001
**Sex** *							
Female	128 (30.8)	77 (35.3)	0.25	68 (33.0)	0.58	45 (22.1)	0.022
Male	287 (69.2)	141 (64.7)		138 (67.0)		159 (77.9)	
**BMI**, mean ± SD	22.4±2.6	21.9±2.8	0.035	21.4±2.7	<0.001	21.5±2.7	<0.001
**Education**							
Illiterate	73 (17.6)	83 (38.6)	<0.001	66 (32.0)	<0.001	44 (21.6)	0.19
Primary school	142 (34.2)	101 (47.0)		107 (51.9)		77 (37.8)	
Middle school or higher	200 (48.2)	31 (14.4)		33 (16.0)		83 (40.7)	
**Smoking**							
Never	217 (52.4)	94 (44.6)	0.063	92 (45.8)	0.12	85 (44.3)	0.062
Ever	197 (47.6)	117 (55.5)		109 (54.2)		107 (55.7)	
pack-years, mean ± SD	23.7±15.6	28.9±21.9	0.030	27.7±19.2	0.068	21.2±14.0	0.20
**Alcohol drinking**							
Never	207(50.2)	116 (55.0)	0.015	111 (55.2)	0.70	87 (45.3)	0.13
Occasionally	72(17.5)	18 (8.5)		31 (15.4)		29(15.1)	
Often	75(18.2)	37 (17.5)		32 (15.9)		51(26.6)	
Everyday	58(14.1)	40 (19.0)		27 (13.4)		25(13.0)	
**H. ** ***pylori*** ** CagA status**							
+	251 (68.8)			130 (64.7)	0.32		
−	114 (31.2)			71 (35.3)			
**HBsAg status**							
+	102 (24.6)					132 (64.7)	<0.001
−	312 (75.4)					72 (35.3)	
**Anti-HCV status**							
+	12 (2.9)					18 (9.0)	0.001
−	403 (97.1)					183 (91.0)	
**Plasma AFB1-albumin adduct levels** (qunitile)							
1	75 (19.9)					26 (14.4)	0.054
2	76 (20.2)					36 (19.9)	
3	75 (19.9)					36 (19.9)	
4	76 (20.2)					28 (15.5)	
5	75 (19.9)					55 (30.4)	

*: Matching factors.

† : Two-sided *P*-values derived from t-tests for continuous variables and χ2 tests for categorical variables comparing cancer cases to controls.

**Figure 1:**
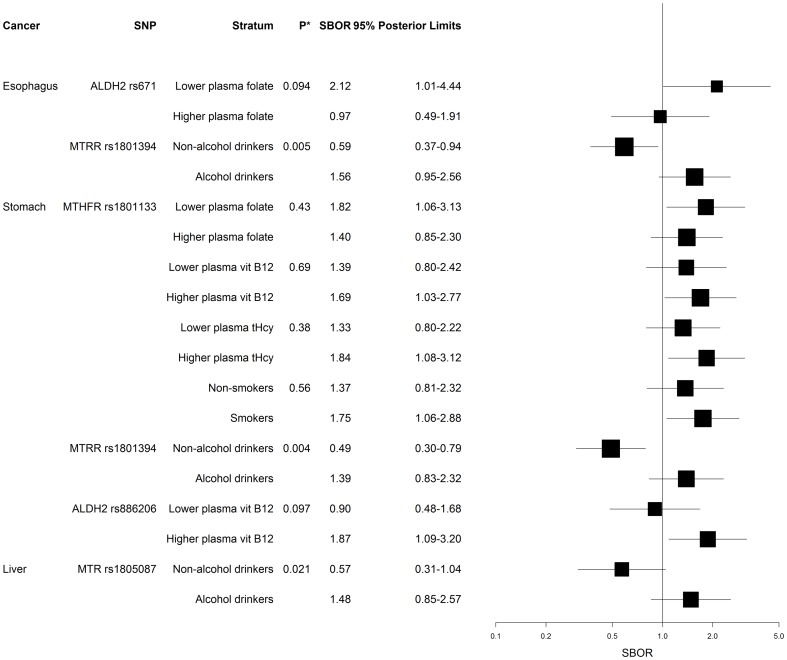
Selected semi-Bayes stratum-specific associations. Selected semi-Bayes stratum-specific associations between SNPs in *MTHFR*, *MTR*, *MTRR*, *DNMT1*, *ALDH2*, and upper GI cancer susceptibility, by plasma levels of micronutrients (folate, vitamin B12, and homocysteine) and environmental factors (smoking, alcohol drinking, H. *pylori* CagA status, HBsAg status, and plasma AFB1-albumin adduct levels). Semi-Bayes adjusted ORs (SBOR) and 95% posterior limits were under dominant genetic models, except for the SBOR relating *ALDH2* rs671to esophageal cancer, where recessive genetic model was used. P* denotes *P*-value for homogeneity test.

Table 2 presents the SB odds-ratio estimates (SBOR) for each SNP-cancer association of the eight SNPs; Table S1, S2, S3, S4, S5, S6 shows stratified associations and Figure 1 summarizes selected results. Genotype distributions among controls appeared compatible with Hardy-Weinberg equilibrium, except possibly for *DNMT1* rs2228612, which had *P* = 0.010, below the traditional alpha level of 0.05, but larger than the Bonferroni-adjusted alpha level of 0.05/8 = 0.006 (testing all eight SNPs). However, we note that matching may bias controls away from equilibrium if the matching factors are associated with both the SNPs and cancer.

**Table 2:** Associations between SNPs of MTHFR, MTR, MTRR, DNMT1, and ALDH2 genes and cancers of the esophagus, stomach, and liver.

	Esophageal Cancer					Stomach Cancer					Liver Cancer				
	Ca/Co	SBOR^1^ (95% posterior limit)	One-sided*P* *	SBOR^2^ (95% posterior limit)	One-sided*P* *	Ca/Co	SBOR^1^ (95% posterior limit)	One-sided *P* *	SBOR^2^ (95% posterior limit)	One- sided *P* *	Ca/Co	SBOR^1^ (95% posterior limit)	One- sided *P* *	SBOR^2^ (95% posterior limit)	One- sided *P* *
**MTHFR**															
**rs1801133**															
CC	65/135	1.00		1.00		50/135	1.00		1.00		50/135	1.00		1.00	
CT	105/199	1.15 (0.79, 1.65)	0.24	1.19 (0.80, 1.77)	0.20	106/199	1.38 (0.94, 2.02)	0.050	1.53 (1.00, 2.33)	0.024	114/199	1.46 (1.00, 2.13)	0.026	1.57 (1.02, 2.42)	0.021
TT	32/57	1.13 (0.69, 1.86)	0.32	1.35 (0.78, 2.32)	0.14	38/57	1.66 (1.02, 2.71)	0.021	2.26 (1.30, 3.91)	0.002	30/57	1.31(0.78, 2.19)	0.15	1.17 (0.64, 2.12)	0.31
*P*_trend_ †		0.50		0.21			0.021		0.001			0.12		0.24	
Dominant		1.15 (0.81, 1.64)	0.22	1.25 (0.85, 1.84)	0.13		1.49 (1.03, 2.16)	0.018	1.79 (1.18, 2.71)	0.003		1.46 (1.00, 2.12)	0.024	1.51 (0.98, 2.32)	0.029
Recessive		1.05 (0.67, 1.66)	0.41	1.23 (0.74, 2.03)	0.21		1.38 (0.90, 2.14)	0.072	1.78 (1.09, 2.91)	0.011		1.05 (0.66, 1.67)	0.41	0.91 (0.53, 1.56)	0.37
**MTR**															
**rs1805087**															
A/A	161/310	1.00		1.00		142/310	1.00		1.00		149/310	1.00		1.00	
A/G	35/76	0.90 (0.58, 1.37)	0.31	0.79 (0.50, 1.27)	0.17	46/76	1.31 (0.87, 1.96)	0.096	1.16 (0.74, 1.83)	0.26	40/76	1.03 (0.68, 1.56)	0.45	0.92 (0.57, 1.47)	0.36
G/G	4/0	–		–		1/0	–		–		1/0	–		–	
Dominant		1.01 (0.66, 1.52)	0.49	0.90 (0.57, 1.42)	0.32		1.34 (0.90, 2.00)	0.078	1.19 (0.76, 1.87)	0.22		1.05 (0.69, 1.59)	0.41	0.93 (0.58, 1.49)	0.38
**MTRR**															
**rs1532268**															
C/C	145/283	1.00		1.00		144/283	1.00		1.00		149/283	1.00		1.00	
C/T	47/94	0.99 (0.67, 1.46)	0.47	1.16 (0.76, 1.78)	0.25	44/94	0.96 (0.65, 1.43)	0.42	0.98 (0.63, 1.53)	0.47	39/94	0.77 (0.51, 1.15)	0.10	0.83 (0.52, 1.32)	0.21
T/T	11/11	1.80 (0.86, 3.80)	0.060	1.96 (0.89, 4.31)	0.047	5/11	1.08 (0.45, 2.56)	0.43	1.05 (0.43, 2.57)	0.46	4/11	0.75 (0.31, 1.82)	0.27	1.09 (0.42, 2.82)	0.43
*P*_trend_ †		0.27		0.070			0.98		0.97			0.14		0.61	
Dominant		1.11 (0.77, 1.61)	0.29	1.32 (0.88, 1.98)	0.089		0.98 (0.67, 1.44)	0.45	1.00 (0.65, 1.52)	0.49		0.75 (0.50, 1.11)	0.075	0.85 (0.54, 1.34)	0.25
Recessive		1.81 (0.86, 3.80)	0.059	1.91 (0.87, 4.19)	0.052		1.09 (0.46, 2.58)	0.43	1.06 (0.43, 2.57)	0.45		0.78 (0.33, 1.89)	0.30	1.12 (0.43, 2.90)	0.41
**rs1801394**															
A/A	117/204	1.00		1.00		119/204	1.00		1.00		114/204	1.00		1.00	
A/G	74/149	0.88 (0.62, 1.25)	0.24	0.92 (0.63, 1.35)	0.34	63/149	0.75 (0.53, 1.08)	0.063	0.74 (0.49, 1.11)	0.070	64/149	0.78 (0.54, 1.12)	0.092	0.74 (0.49, 1.12)	0.078
G/G	10/25	0.81 (0.42, 1.59)	0.27	0.95 (0.47, 1.93)	0.44	9/25	0.76 (0.38, 1.50)	0.21	0.77 (0.37, 1.60)	0.24	13/25	0.93 (0.49, 1.75)	0.41	1.39 (0.70, 2.78)	0.17
*P*_trend_ †		0.36		0.70			0.10		0.13			0.30		0.87	
Dominant		0.86 (0.61, 1.21)	0.20	0.92 (0.64, 1.34)	0.33		0.74 (0.52, 1.05)	0.046	0.73 (0.49, 1.07)	0.055		0.80 (0.56, 1.13)	0.10	0.83 (0.56, 1.24)	0.19
Recessive		0.85 (0.44, 1.64)	0.31	0.97 (0.48, 1.96)	0.47		0.82 (0.41, 1.62)	0.29	0.84 (0.41, 1.74)	0.32		1.00 (0.54, 1.88)	0.50	1.53 (0.78, 3.03)	0.11
**DNMT1**															
**rs2228612**															
A/A	52/100	1.00		1.00		43/100	1.00		1.00		48/100	1.00		1.00	
A/G	56/200	0.58 (0.38, 0.88)	0.006	0.52 (0.33, 0.82)	0.003	72/200	0.84 (0.55, 1.28)	0.21	0.82 (0.51, 1.31)	0.20	74/200	0.79 (0.52, 1.19)	0.13	0.79 (0.49, 1.28)	0.17
G/G	29/57	1.03 (0.61, 1.73)	0.46	0.96 (0.55, 1.68)	0.44	28/57	1.11 (0.65, 1.89)	0.36	1.12 (0.62, 2.04)	0.35	36/57	1.33 (0.80, 2.22)	0.13	1.35 (0.76, 2.42)	0.15
*P*_trend_ †		0.57		0.37			0.88		0.88			0.42		0.42	
Dominant		0.67 (0.45, 1.00)	0.026	0.60 (0.39, 0.94)	0.012		0.90 (0.59, 1.36)	0.31	0.89 (0.56, 1.41)	0.31		0.92 (0.62, 1.37)	0.34	0.93 (0.58, 1.50)	0.39
Recessive		1.38 (0.86, 2.21)	0.092	1.35 (0.81, 2.25)	0.13		1.22 (0.76, 1.97)	0.20	1.26 (0.74, 2.15)	0.20		1.53 (0.97, 2.40)	0.033	1.54 (0.92, 2.59)	0.051
**ALDH2**															
**rs671**															
G/G	118/213	1.00		1.00		108/213	1.00		1.00		109/213	1.00		1.00	
A/G	60/160	0.70 (0.49, 1.01)	0.029	0.76 (0.51, 1.13)	0.088	76/160	0.97 (0.68, 1.38)	0.44	1.06 (0.71, 1.57)	0.39	74/160	0.91 (0.64, 1.30)	0.31	1.05 (0.70, 1.58)	0.41
A/A	25/20	1.97 (1.11, 3.49)	0.010	1.61 (0.86, 3.00)	0.066	12/20	1.13 (0.58, 2.21)	0.36	0.99 (0.48, 2.03)	0.49	10/20	1.03 (0.51, 2.06)	0.47	0.96 (0.45, 2.06)	0.46
*P*_trend_ †		0.49		0.67			0.88		0.88			0.77		0.94	
Dominant		0.88 (0.63, 1.24)	0.24	0.90 (0.62, 1.31)	0.29		1.00 (0.71, 1.40)	0.49	1.05 (0.71, 1.54)	0.41		0.93 (0.66, 1.31)	0.33	1.04 (0.70, 1.54)	0.43
Recessive		2.20 (1.25, 3.86)	0.003	1.76 (0.96, 3.24)	0.035		1.14 (0.59, 2.21)	0.35	0.97 (0.48, 1.97)	0.47		1.06 (0.53, 2.10)	0.44	0.95 (0.45, 2.01)	0.44
**rs2238151**															
C/C	173/293	1.00		1.00		151/293	1.00		1.00		166/293	1.00		1.00	
C/T	11/34	0.66 (0.35, 1.22)	0.093	0.66 (0.34, 1.29)	0.11	18/34	1.08 (0.61, 1.89)	0.40	1.21 (0.65, 2.23)	0.28	7/34	0.44 (0.22, 0.87)	0.009	0.51 (0.23, 1.11)	0.045
T/T	0/1	–		–		0/1	–		–		1/1	1.15 (0.33, 4.03)	0.41	1.43 (0.39, 5.33)	0.30
*P*_trend_ †		–		–			–		–			0.045		0.29	
Dominant		0.64 (0.35, 1.19)	0.079	0.65 (0.33, 1.28)	0.11		1.05 (0.60, 1.83)	0.43	1.20 (0.65, 2.21)	0.29		0.47 (0.24, 0.92)	0.013	0.58 (0.27, 1.25)	0.082
Recessive		–		–			–		–			1.16 (0.33, 4.07)	0.41	1.44 (0.39, 5.37)	0.29
**rs886205**															
G/G	173/315	1.00		1.00		150/315	1.00		1.00		149/315	1.00		1.00	
A/G	29/69	0.81 (0.51, 1.26)	0.17	0.78 (0.48, 1.28)	0.17	41/69	1.23 (0.81, 1.87)	0.17	1.47 (0.92, 2.34)	0.054	40/69	1.19 (0.78, 1.81)	0.21	1.38 (0.85, 2.24)	0.096
A/A	2/4	1.07 (0.36, 3.18)	0.45	1.00 (0.33, 3.08)	0.50	2/4	1.10 (0.37, 3.30)	0.43	1.18 (0.39, 3.65)	0.38	2/4	1.00 (0.34, 2.96)	0.50	1.27 (0.40, 4.10)	0.34
*P*_trend_ †		0.45		0.39			0.32		0.098			0.47		0.14	
Dominant		0.82 (0.53, 1.28)	0.19	0.79 (0.49, 1.29)	0.17		1.23 (0.82, 1.86)	0.16	1.48 (0.93, 2.34)	0.047		1.18 (0.78, 1.79)	0.22	1.41 (0.88, 2.28)	0.076
Recessive		1.08 (0.36, 3.23)	0.44	1.02 (0.33, 3.13)	0.49		1.08 (0.36, 3.24)	0.44	1.16 (0.38, 3.55)	0.40		0.98 (0.33, 2.92)	0.49	1.25 (0.39, 4.03)	0.35

1 : Semi-Bayes odds ratio (SBOR) adjusted for age (5-year categories and deviation from stratum mean) and sex.

2 : SBOR further adjusted for residency (city, rural), alcohol drinking frequency, smoking pack-years, BMI, education, H. *pylori* infection (in stomach cancer analyses), HBsAg (in liver cancer analyses), and plasma AFB1 levels (in liver cancer analyses), based on the assumption that genotype did not affect any of these variables.

*: One-sided semi-Bayes *P*-values; the posterior probability that the point estimate is on the wrong side of the null [26], [27].

† : *P*-value from Chi-Square test for trend.

We have previously reported positive associations of the T allele of *MTHFR* rs1801133 with stomach and liver cancer [11], [12]. In the present analysis, these associations remained apparent after confounder adjustment and SB shrinkage (any T versus C/C, fully adjusted SBOR: 1.79, 95% posterior limits: 1.18, 2.71 for stomach cancer; SBOR: 1.51, 95% posterior limits: 0.98, 2.32 for liver cancer). In stratified SB analyses, the association between *MTHFR* rs1801133 and stomach cancer appeared stronger among individuals who had lower plasma folate levels, higher plasma vitamin B12 or tHcy levels, and among smokers (Figure 1). There was no clear association of *MTHFR* rs1801133 with esophageal cancer (Table 2 and Tables S1, S2, S3, S4, S5).

While there was no clear overall association between SNPs in *MTR* and *MTRR* and any cancer in main effect analyses (Table 2), heterogeneity of association was suggested in stratified analyses on alcohol consumption, including associations of *MTR* rs1805087 with liver cancer (homogeneity *P* = 0.021), and *MTRR* rs1801394 with both esophageal (homogeneity *P* = 0.005) and stomach cancer (homogeneity *P* = 0.004). While G allele carriers of *MTR* rs1805087 were inversely associated with liver cancer among non-drinkers (SBOR: 0.57, 95% posterior limits: 0.31, 1.04), they were positively associated with liver cancer among drinkers (SBOR: 1.48, 95% posterior limits: 0.85, 2.57) (Figure 1). Similarly, G allele carriers of *MTRR* rs1801394 were inversely associated with esophageal and stomach cancer among non-drinkers (SBOR: 0.59, 95% posterior limits: 0.37, 0.94 for esophageal cancer; SBOR: 0.49, 95% posterior limits: 0.30, 0.79 for stomach cancer) but positively associated with cancer among drinkers (SBOR: 1.56, 95% posterior limits: 0.95, 2.56 for esophageal cancer; SBOR: 1.39, 95% posterior limits: 0.83, 2.32 for stomach cancer) (Figure 1).

For *DNMT1* polymorphism, rs2228612 was inversely associated with esophageal cancer in the dominant genetic model (any G versus A/A, SBOR: 0.60, 95% posterior limits: 0.39, 0.94) (Table 2). Among three *ALDH2* SNPs, rs671 was associated with esophageal cancer in the recessive genetic model (A/A versus any G, SBOR: 1.76, 95% posterior limits: 0.96, 3.24). In stratified adjusted analyses, *ALDH2* rs671 appeared associated with esophageal cancer among individuals with lower plasma folate levels (A/A versus any G, SBOR: 2.12, 95% posterior limits: 1.01, 4.44) (Figure 1). The *ALDH2* rs2238151 appeared inversely associated with liver cancer when comparing T allele carriers to those with the C/C genotype (age and sex-adjusted SBOR: 0.47, 95% posterior limits: 0.24, 0.92). While we did not find associations between *ALDH2* rs886205 and cancer susceptibility in main effect analyses, stratum-specific SBOR suggested that *ALDH2* rs886205 was positively associated with stomach cancer among participants with higher plasma vitamin B12 levels (SBOR: 1.87, 95% posterior limits: 1.09, 3.20) (Figure 1).

Except for analysis on single SNP models, we also did joint SNPs analysis by including all of the 8 SNPs in a model (Table 3). The results from joint SNPs analysis suggested similar associations as in the single SNP models, but the 95% posterior intervals were wider.

**Table 3:** Associations between SNPs of MTHFR, MTR, MTRR, DNMT1, and ALDH2 genes and cancers of the esophagus, stomach, and liver –the comparison between results from single SNP model^1^ and joint SNPs model^2^.

	Esophageal Cancer					Stomach Cancer					Liver Cancer				
	Ca/Co	SBOR^1^ (95% posterior limit)	One-sided*P* *	SBOR^2^ (95% posterior limit)	One-sided*P* *	Ca/Co	SBOR^1^ (95% posterior limit)	One-sided *P* *	SBOR^2^ (95% posterior limit)	One- sided *P* *	Ca/Co	SBOR^1^ (95% posterior limit)	One- sided *P* *	SBOR^2^ (95% posterior limit)	One- sided *P* *
**MTHFR**															
**rs1801133**															
CC	65/135	1.00		1.00		50/135	1.00		1.00		50/135	1.00		1.00	
CT	105/199	1.19 (0.80, 1.77)	0.20	1.20 (0.73, 1.96)	0.24	106/199	1.53 (1.00, 2.33)	0.024	1.33 (0.79, 2.21)	0.14	114/199	1.57 (1.02, 2.42)	0.021	1.34 (0.80, 2.25)	0.14
TT	32/57	1.35 (0.78, 2.32)	0.14	1.72 (0.91, 3.27)	0.048	38/57	2.26 (1.30, 3.91)	0.002	2.15 (1.13, 4.08)	0.010	30/57	1.17 (0.64, 2.12)	0.31	1.07 (0.52, 2.20)	0.42
*P*_trend_ †		0.21		0.033			0.001		0.003			0.24		0.18	
Dominant		1.25 (0.85, 1.84)	0.13	1.37 (0.85, 2.21)	0.10		1.79 (1.18, 2.71)	0.003	1.64 (1.01, 2.66)	0.022		1.51 (0.98, 2.32)	0.029	1.40 (0.88, 2.24)	0.078
Recessive		1.23 (0.74, 2.03)	0.21	1.55 (0.87, 2.75)	0.069		1.78 (1.09, 2.91)	0.011	1.93 (1.11, 3.35)	0.009		0.91 (0.53, 1.56)	0.37	1.00 (0.54, 1.83)	0.49
**MTR**															
**rs1805087**															
A/A	161/310	1.00		1.00		142/310	1.00		1.00		149/310	1.00		1.00	
A/G	35/76	0.79 (0.50, 1.27)	0.17	0.83 (0.48, 1.45)	0.26	46/76	1.16 (0.74, 1.83)	0.26	1.23 (0.73, 2.09)	0.22	40/76	0.92 (0.57, 1.47)	0.36	0.94 (0.54, 1.63)	0.41
G/G	4/0	–		–		1/0	–		–		1/0	–		–	
Dominant		0.90 (0.57, 1.42)	0.32	0.94 (0.55, 1.62)	0.42		1.19 (0.76, 1.87)	0.22	1.30 (0.78, 2.15)	0.16		0.93 (0.58, 1.49)	0.38	1.20 (0.73, 1.98)	0.23
**MTRR**															
**rs1532268**															
C/C	145/283	1.00		1.00		144/283	1.00		1.00		149/283	1.00		1.00	
C/T	47/94	1.16 (0.76, 1.78)	0.25	0.99 (0.57, 1.70)	0.48	44/94	0.98 (0.63, 1.53)	0.47	1.01 (0.58, 1.77)	0.48	39/94	0.83 (0.52, 1.32)	0.21	1.03 (0.59, 1.81)	0.46
T/T	11/11	1.96 (0.89, 4.31)	0.047	1.61 (0.61, 4.25)	0.17	5/11	1.05 (0.43, 2.57)	0.46	0.83 (0.25, 2.70)	0.38	4/11	1.09 (0.42, 2.82)	0.43	1.58 (0.54, 4.63)	0.20
*P*_trend_ †		0.070		0.21			0.97		0.49			0.61		0.50	
Dominant		1.32 (0.88, 1.98)	0.089	1.12 (0.67, 1.87)	0.34		1.00 (0.65, 1.52)	0.49	1.06 (0.63, 1.80)	0.41		0.85 (0.54, 1.34)	0.25	0.97 (0.59, 1.60)	0.46
Recessive		1.91 (0.87, 4.19)	0.052	1.70 (0.66, 4.40)	0.14		1.06 (0.43, 2.57)	0.45	0.87 (0.27, 2.81)	0.41		1.12 (0.43, 2.90)	0.41	1.41 (0.51, 3.86)	0.25
**rs1801394**															
A/A	117/204	1.00		1.00		119/204	1.00		1.00		114/204	1.00		1.00	
A/G	74/149	0.92 (0.63, 1.35)	0.34	0.84 (0.51, 1.36)	0.24	63/149	0.74 (0.49, 1.11)	0.070	0.66 (0.40, 1.08)	0.049	64/149	0.74 (0.49, 1.12)	0.078	0.79 (0.47, 1.31)	0.18
G/G	10/25	0.95 (0.47, 1.93)	0.44	1.16 (0.52, 2.59)	0.36	9/25	0.77 (0.37, 1.60)	0.24	0.98 (0.41, 2.34)	0.49	13/25	1.39 (0.70, 2.78)	0.17	1.60 (0.72, 3.55)	0.12
*P*_trend_ †		0.70		0.50			0.13		0.075			0.87		0.33	
Dominant		0.92 (0.64, 1.34)	0.33	0.89 (0.56, 1.43)	0.32		0.73 (0.49, 1.07)	0.055	0.65 (0.41, 1.03)	0.034		0.83 (0.56, 1.24)	0.19	0.95 (0.61, 1.49)	0.42
Recessive		0.97 (0.48, 1.96)	0.47	1.35 (0.62, 2.92)	0.23		0.84 (0.41, 1.74)	0.32	1.09 (0.48, 2.47)	0.42		1.53 (0.78, 3.03)	0.11	1.49 (0.71, 3.13)	0.14
**DNMT1**															
**rs2228612**															
A/A	52/100	1.00		1.00		43/100	1.00		1.00		48/100	1.00		1.00	
A/G	56/200	0.52 (0.33, 0.82)	0.003	0.52 (0.31, 0.85)	0.005	72/200	0.82 (0.51, 1.31)	0.20	0.81 (0.48, 1.36)	0.21	74/200	0.79 (0.49, 1.28)	0.17	0.69 (0.40, 1.18)	0.088
G/G	29/57	0.96 (0.55, 1.68)	0.44	0.87 (0.47, 1.58)	0.32	28/57	1.12 (0.62, 2.04)	0.35	1.06 (0.55, 2.04)	0.43	36/57	1.35 (0.76, 2.42)	0.15	1.25 (0.66, 2.35)	0.25
*P*_trend_ †		0.37		0.12			0.88		0.48			0.42		0.32	
Dominant		0.60 (0.39, 0.94)	0.012	0.58 (0.36, 0.94)	0.013		0.89 (0.56, 1.41)	0.31	0.90 (0.55, 1.48)	0.34		0.93 (0.58, 1.50)	0.39	0.82 (0.51, 1.32)	0.21
Recessive		1.35 (0.81, 2.25)	0.13	1.20 (0.69, 2.09)	0.25		1.26 (0.74, 2.15)	0.20	1.19 (0.69, 2.07)	0.26		1.54 (0.92, 2.59)	0.051	1.62 (0.97, 2.71)	0.033
**ALDH2**															
**rs671**															
G/G	118/213	1.00		1.00		108/213	1.00		1.00		109/213	1.00		1.00	
A/G	60/160	0.76 (0.51, 1.13)	0.088	0.78 (0.48, 1.27)	0.16	76/160	1.06 (0.71, 1.57)	0.39	0.99 (0.61, 1.62)	0.49	74/160	1.05 (0.70, 1.58)	0.41	1.04 (0.63, 1.70)	0.44
A/A	25/20	1.61 (0.86, 3.00)	0.066	1.10 (0.52, 2.31)	0.40	12/20	0.99 (0.48, 2.03)	0.49	1.12 (0.51, 2.47)	0.39	10/20	0.96 (0.45, 2.06)	0.46	0.80 (0.34, 1.92)	0.31
*P*_trend_ †		0.67		0.31			0.88		0.49			0.94		0.33	
Dominant		0.90 (0.62, 1.31)	0.29	0.80 (0.51, 1.26)	0.17		1.05 (0.71, 1.54)	0.41	1.00 (0.63, 1.58)	0.50		1.04 (0.70, 1.54)	0.43	0.94 (0.61, 1.47)	0.40
Recessive		1.76 (0.96, 3.24)	0.035	1.16 (0.56, 2.37)	0.35		0.97 (0.48, 1.97)	0.47	1.01 (0.48, 2.14)	0.49		0.95 (0.45, 2.01)	0.44	0.84 (0.37, 1.87)	0.33
**rs2238151**															
C/C	173/293	1.00		1.00		151/293	1.00		1.00		166/293	1.00		1.00	
C/T	11/34	0.66 (0.34, 1.29)	0.11	0.81 (0.35, 1.86)	0.31	18/34	1.21 (0.65, 2.23)	0.28	1.00 (0.45, 2.22)	0.50	7/34	0.51 (0.23, 1.11)	0.045	0.36 (0.14, 0.92)	0.016
T/T	0/1	–		–		0/1	–				1/1	1.43 (0.39, 5.33)	0.30	1.00 (0.25, 4.00)	0.50
*P*_trend_ †		–		–			–					0.29		0.006	
Dominant		0.65 (0.33, 1.28)	0.11	0.80 (0.35, 1.84)	0.30		1.20 (0.65, 2.21)	0.29	1.08 (0.49, 2.35)	0.43		0.58 (0.27, 1.25)	0.082	0.31 (0.13, 0.75)	0.005
Recessive		–		–			–					1.44 (0.39, 5.37)	0.29	1.00 (0.25, 4.00)	0.50
**rs886205**															
G/G	173/315	1.00		1.00		150/315	1.00		1.00		149/315	1.00		1.00	
A/G	29/69	0.78 (0.48, 1.28)	0.17	1.05 (0.55, 2.00)	0.44	41/69	1.47 (0.92, 2.34)	0.054	1.76 (0.93, 3.33)	0.041	40/69	1.38 (0.85, 2.24)	0.096	1.41 (0.75, 2.66)	0.14
A/A	2/4	1.00 (0.33, 3.08)	0.50	0.82 (0.25, 2.69)	0.37	2/4	1.18 (0.39, 3.65)	0.38	0.65 (0.19, 2.23)	0.25	2/4	1.27 (0.40, 4.10)	0.34	1.07 (0.31, 3.76)	0.46
*P*_trend_ †		0.39		0.37			0.098		0.31			0.14		0.17	
Dominant		0.79 (0.49, 1.29)	0.17	0.99 (0.52, 1.86)	0.48		1.48 (0.93, 2.34)	0.047	1.38 (0.76, 2.51)	0.15		1.41 (0.88, 2.28)	0.076	1.34 (0.76, 2.37)	0.16
Recessive		1.02 (0.33, 3.13)	0.49	0.85 (0.26, 2.75)	0.39		1.16 (0.38, 3.55)	0.40	0.70 (0.20, 2.40)	0.28		1.25 (0.39, 4.03)	0.35	0.93 (0.28, 3.04)	0.45

1 : Single SNP model: models included one SNP only; Semi-Bayes odds ratio (SBOR) adjusted for age (5-year categories and deviation from stratum mean), sex, residency (city, rural), alcohol drinking frequency, smoking pack-years, BMI, education, H. *pylori* infection (in stomach cancer analyses), HBsAg (in liver cancer analyses), and plasma AFB1 levels (in liver cancer analyses), based on the assumption that genotype did not affect any of these variables.

2 : Joint SNPs model: models included all of the 8 SNPs; SBOR adjusted for the same set of covariates. All of the SNPs combined together were under the same genetic models (genotype-specific, log-additive, dominant, or recessive).

*: One-sided semi-Bayes *P*-values; the posterior probability that the point estimate is on the wrong side of the null [26], [27].

† : *P*-value from Chi-Square test for trend.

The analysis on PRS suggested roughly a doubling of odds for esophageal and liver cancers among individuals in the highest PRS category compared to those in the lowest category (SBOR: 2.06; 95% posterior limits: 1.13, 3.77 for esophageal cancer and SBOR: 2.09, 95% posterior limits: 1.05, 4.17 for liver cancer), with somewhat less consistency across categories for stomach cancer. In the continuous PRS analysis, the results suggested a doubling of odds for these three upper GI cancers with one unit (in log OR) increase of PRS (Table 4). We caution however that PRS analyses do not account for the score construction from the data, and thus may overestimate effects and underestimate variability in the resulting estimates.

**Table 4:** Association between polygenetic risk score and cancers of the esophagus, stomach, and liver¶.

Polygenetic risk score (8 SNPs)	Esophageal cancer			Stomach cancer			Liver cancer		
	Ca/Co	SBOR†	One-sided *P* *	Ca/Co	SBOR†	One-sided *P* *	Ca/Co	SBOR†	One-sided *P* *
**Equal-spaced categories**									
Lowest	20/65	1.00		37/131	1.00		27/64	1.00	
Middle	65/165	1.21 (0.70, 2.08)	0.25	74/123	2.07 (1.28, 3.35)	0.001	88/194	0.95 (0.55, 1.64)	0.43
Highest	41/57	2.06 (1.13, 3.77)	0.010	14/33	1.55 (0.77, 3.11)	0.11	27/29	2.09 (1.05, 4.17)	0.019
**Continuous (1 unit increase in PRS)**		2.13 (1.23, 3.69)	0.004		2.37 (1.34, 4.19)	0.002		2.17 (1.11, 4.25)	0.012

† : Semi-Bayes odds ratio (SBOR) adjusted for age (5-year categories and deviation from stratum mean), sex, residency (city, rural), alcohol drinking frequency, smoking pack-years, BMI, education, H. *pylori* infection (in stomach cancer analyses), HBsAg (in liver cancer analyses), and plasma AFB1 levels (in liver cancer analyses), based on the assumption that genotype did not affect any of these variables.

*: One-sided semi-Bayes *P*-values; the posterior probability that the point estimate is on the wrong side of the null [26], [27].

¶ : Polygenetic risk score (PRS) was calculated only among subjects with complete data on all 8 SNPs.

## Discussion

We examined the associations between eight SNPs in genes involved in the one-carbon metabolic pathway and susceptibility of esophageal, stomach, and liver cancers in a Chinese population. After applying SB shrinkage methods and controlling for potential confounders, we observed that any T genotype of *MTHFR* rs1801133 was positively associated with both stomach and liver cancer. We also found an inverse association between the variant G allele of *DNMT1* rs2228612 and esophageal cancer. In addition, our study suggested potential OR variations across strata of alcohol consumption, including associations of *MTRR* rs1801394 with esophageal and stomach cancer, and *MTR* rs1805087 with liver cancer. The odds for upper GI cancers were roughly doubled for Chinese participants with one unit (in log OR) increase of PRS.

In one-carbon metabolism, MTHFR irreversibly catalyzes the conversion of 5,10-methylenetetrahydrofolate (5,10-methyleneTHF) to 5-methyltetrahydrofolate (5-methylTHF). The 5,10-methyleneTHF is essential in purine and thymidilate synthesis, and 5-methylTHF is a co-substrate for remethylation of homocysteine to methionine, which is further converted to SAM for methylation reactions [5]. The *MTHFR* C677T (rs1801133) polymorphism, which results in an alanine to valine substitution, leads to reduced MTHFR enzyme activity [29], decreased 5-methylTHF and an accumulation of 5,10-methyleneTHF in red blood cells [30].

Low MTHFR activity is associated with increase cancer risk due to low blood 5-methylTHF and impaired DNA methylation. Conversely, it could reduce cancer risk by increasing the availability of 5,10-methyleneTHF for normal DNA synthesis and preventing uracil misincorporation and chromosomal breakage [5]. Although evidence in support of these hypotheses is weak and inconsistent [5], an *in vitro* study suggested that the effect of *MTHFR* rs1801133 on DNA stability and methylation is site-specific and may depend on folate availability [31]. When folate supply is adequate or high, the T allele of *MTHFR* is associated with increased genomic DNA methylation in colon cancer cells, but decreased DNA methylation in breast cancer cells. When folate supply is limited, this variant is associated with decreased and unchanged DNA methylation in colon and breast cancer cells, respectively [31]. Uracil misincorporation is decreased in colon cancer cells expressing the *MTHFR* T allele, and increased in breast cancer cells expressing the same variant [31]. This site-specific difference may partly explain the difference in cancer risk associated with the *MTHFR* rs1801133 polymorphism [4]. In epidemiologic studies, the T allele appears to decrease the risk of colorectal and breast cancers [32], [33], but increase the risk of cancers of the esophagus, stomach, liver, bladder, cervix uteri, and lung [2]–[4], [34]–[36].

In the present analysis using SB shrinkage, we confirmed our previous findings of positive associations between the T allele of *MTHFR* rs1801133 and cancers of the stomach and liver in this Taixing population [11], [12], implying that the disturbance of DNA methylation resulting from this variant plays a major role in stomach and liver carcinogenesis. Recent meta-analyses reported similar associations (T/T versus C/C, OR: 1.40, 95% CI: 1.19–1.66 for stomach cancer; OR: 1.21, 95% CI: 0.95–1.56 for liver cancer) [3], [4]. In addition, Zacho et al. [4] reported a larger association between *MTHFR* rs1801133 and stomach cancer among study populations without folic acid fortification (OR: 1.60, 95% CI: 1.36–1.88), as compared to those with fortification (OR: 1.15, 95% CI: 0.81–1.63), which is similar to our finding of a stronger association among individuals with lower plasma folate levels. For esophageal cancer, our data suggested an increased risk among *MTHFR* rs1801133 T allele carriers (any T vs. C/C, SBOR: 1.25, 95% posterior limits: 0.85, 1.84), which is consistent with findings from a meta-analysis of 19 studies (C/T versus C/C, OR: 1.47, 95% CI: 1.32–1.63; T/T versus C/C, OR: 1.69, 95% CI: 1.49–1.91) [2].

MTR and MTRR are two other important enzymes involved in one-carbon metabolism. MTR catalyzes the methylation of homocysteine to methionine. *MTR* A2756G (rs1805087), a common SNP leading to the substitution of aspartic acid with glycine, has been largely studied. However, no apparent associations have been observed with cancer at the following sites: lung, prostate, head and neck, bladder, esophagus, stomach, breast, or colon and rectal [37]–[46]. MTRR regenerates a functional MTR via reductive methylation. Two common polymorphisms, *MTRR* A66G (rs1801394, converts isoleucine to methionine) and C524T (rs1532268, changes serine to leucine), have been indicated to regenerate MTR less efficiently [47]. G allele carriers of *MTRR* rs1801394 have been associated with increased risk for hepatocellular carcinoma (HCC) [48]. Conversely, associations are inconsistent with other malignancies, including esophageal squamous cell carcinoma (ESCC), stomach cancer, and colorectal cancer [37], [44], [49]–[53]. Most studies that have investigated *MTRR* rs1532268 reported no associations with colorectal, gastric, breast, and lung cancer [44], [51], [53]–[56]. One should bear in mind however that apparent inconsistencies and reports of no association may only reflect expected variation in *P*-values (“statistical significance”) rather than any real conflicts.

Our study observed odds-ratio variation of the associations between these *MTR*/*MTRR* polymorphisms and upper GI cancers across alcohol consumption, even after conservative SB shrinkage. Alcohol consumption appeared to have modified odds-ratios relating *MTR* rs1805087 to liver cancer, and *MTRR* rs1801394 to esophageal and stomach cancer. G allele carriers of these two SNPs were positively associated with cancer among drinkers, and inversely associated with cancer among non-drinkers. Matsuo et al., observed a similar OR variation [57]: G/G genotype carriers of *MTR* rs1805087 showed higher colorectal cancer risk among alcohol drinkers and lower risk among non-drinkers. Although the functional effect of *MTR/MTRR* polymorphisms has not been established, our results are biologically plausible as alcohol can disrupt one-carbon metabolism by inhibiting folate absorption, suppressing SAM synthesis, and impairing DNA methylation [6]. Alcohol can also cause inhibition of methionine synthase activity [6]. Therefore, it is possible that the variant allele of these two *MTR/MTRR* polymorphisms is protective for the upper GI cancers under the environment without alcohol exposures. However, it becomes deleterious when one-carbon metabolism is disrupted by alcohol and its metabolites.

ALDH2 is involved in alcohol metabolism by oxidizing acetaldehyde, a group 2B human carcinogen, to acetic acid. The *ALDH2* rs671 polymorphism—a well-known variant that occurs exclusively in Asian populations—causes a lower catalytic efficiency of ALDH2, and hence renders lower ability to eliminate acetaldehyde [58]. The *ALDH2* rs671 A allele (slow type) has been associated with increased risk of head and neck cancer, as well as esophageal cancer [59], [60]. Consistent with previous findings, we observed a positive association between the A/A genotype and esophageal cancer in this study, and further reported a stronger association among individuals with lower plasma folate levels. Acetaldehyde also interferes with folate metabolism [6]. It is possible that the deleterious effect associated with rs671 polymorphism is more prominent under the condition of lower folate supply. Another common variation in the *ALDH2* gene— rs886205 with a G to A substitution in the promoter region—has been suggested to be functional. Chou et al., reported that the promoter constructs encoded by the G allele were more active than the A allele in hepatoma cells [61]. The G allele of rs886205 was reported to be associated with increased risk of ESCC [62], [63] but not with stomach [64], [65] and colorectal cancer [66]. It also showed inconsistent results with head and neck cancer [67]–[69]. We observed a positive association between the A allele of rs886205 and stomach cancer among those with higher plasma vitamin B12 levels. Although *ALDH2* rs886205 is suggested to be a functional polymorphism in hepatoma cells [61], further functionality studies are warranted.

There are several limitations due to the case-control design and the multiple comparisons in this study. Because we were not able to recruit all of the identified cases and controls, selection bias may occur if participation is affected by an un-identified factor which is associated with both the SNPs and cancer. On the other hand, cancers of the esophagus, stomach, and liver are fatal, and some patients with late clinical stages at diagnosis were either too ill to participate or passed away. This selection of patients may have resulted in biased estimates if the SNPs under study are associated with disease progression. Also, we collected plasma samples after cancer diagnoses. By stratification on plasma micronutrients, we may have introduced a “collider-stratification bias” if disease states, as well as treatments and/or diet and behavior changes among cancer patients would affect the levels of these biomarkers [70]. However, given that there may be only a weak association between SNPs and plasma micronutrients in one-carbon metabolic pathway [71], we believe that the size of the bias would be small. In addition, we conducted many comparisons and subgroup analyses, which led us to employ semi-Bayes shrinkage estimation to reduce the risk of misleading results. Using these methods, in this Chinese population, several polymorphisms in the one-carbon metabolic pathway appear to be associated with esophageal, stomach, and liver cancer, with heterogeneity across strata of alcohol consumption for the odds ratios relating *MTR*/*MTRR* polymorphisms to these cancers, suggesting potential interactions between alcohol drinking and genes of the one-carbon metabolic pathway. Confirmation of these results and research on the underlying mechanisms are needed.

## Supporting Information

Table S1 **Results of the associations between SNPs of MTHFR, MTR, MTRR, DNMT1, and ALDH2 genes and cancers of esophagus, stomach, and liver, stratified on plasma folate levels.**
(DOC)

Table S2 **Results of the associations between SNPs of MTHFR, MTR, MTRR, DNMT1, and ALDH2 genes and cancers of esophagus, stomach, and liver, stratified on plasma vitamin B12 levels.**
(DOC)

Table S3 **Results of the associations between SNPs of MTHFR, MTR, MTRR, DNMT1, and ALDH2 genes and cancers of esophagus, stomach, and liver, stratified on plasma homocysteine levels.**
(DOC)

Table S4 **Results of the associations between SNPs of MTHFR, MTR, MTRR, DNMT1, and ALDH2 genes and cancers of esophagus, stomach, and liver, stratified on tobacco smoking status.**
(DOC)

Table S5 **Results of the associations between SNPs of MTHFR, MTR, MTRR, DNMT1, and ALDH2 genes and cancers of esophagus, stomach, and liver, stratified on alcohol drinking status.**
(DOC)

Table S6 **Results of the associations between SNPs of MTHFR, MTR, MTRR, DNMT1, and ALDH2 genes and stomach cancer, stratified on H. Pylori infection status, and on liver cancer, stratified on HBsAg status and plasma aflatoxin B1 albumin adduct levels.**
(DOC)

## References

[1] Ferlay J, Soerjomataram I, Ervik M, Dikshit R, Eser S, et al.. (2013) GLOBOCAN 2012 v1.0, Cancer Incidence and Mortality Worldwide. IARC CancerBase no 11. Lyon, France: International Agency for Research on Cancer.

[2] Fang Y, Xiao F, An Z, Hao L (2011) Systematic review on the relationship between genetic polymorphisms of methylenetetrahydrofolate reductase and esophageal squamous cell carcinoma. Asian Pac J Cancer Prev 12: 1861–1866. 22126580

[3] Qin X, Peng Q, Chen Z, Deng Y, Huang S, et al (2013) The association between MTHFR gene polymorphisms and hepatocellular carcinoma risk: a meta-analysis. PLoS One 8: e56070. 23457501 10.1371/journal.pone.0056070 PMC3573065

[4] Zacho J, Yazdanyar S, Bojesen SE, Tybjaerg-Hansen A, Nordestgaard BG (2011) Hyperhomocysteinemia, methylenetetrahydrofolate reductase c.677C>T polymorphism and risk of cancer: cross-sectional and prospective studies and meta-analyses of 75,000 cases and 93,000 controls. Int J Cancer 128: 644–652. 20473868 10.1002/ijc.25375

[5] Duthie SJ (2011) Folate and cancer: how DNA damage, repair and methylation impact on colon carcinogenesis. J Inherit Metab Dis 34: 101–109. 20544289 10.1007/s10545-010-9128-0

[6] Seitz HK, Stickel F (2007) Molecular mechanisms of alcohol-mediated carcinogenesis. Nat Rev Cancer 7: 599–612. 17646865 10.1038/nrc2191

[7] Ghoshal K, Li X, Datta J, Bai S, Pogribny I, et al (2006) A folate- and methyl-deficient diet alters the expression of DNA methyltransferases and methyl CpG binding proteins involved in epigenetic gene silencing in livers of F344 rats. J Nutr 136: 1522–1527. 16702315 10.1093/jn/136.6.1522 PMC2237890

[8] Huang RF, Hsu YC, Lin HL, Yang FL (2001) Folate depletion and elevated plasma homocysteine promote oxidative stress in rat livers. J Nutr 131: 33–38. 11208935 10.1093/jn/131.1.33

[9] Gonda TA, Kim YI, Salas MC, Gamble MV, Shibata W, et al (2012) Folic acid increases global DNA methylation and reduces inflammation to prevent Helicobacter-associated gastric cancer in mice. Gastroenterology 142: 824–833 e827. 22248660 10.1053/j.gastro.2011.12.058

[10] Xiao SD, Meng XJ, Shi Y, Hu YB, Zhu SS, et al (2002) Interventional study of high dose folic acid in gastric carcinogenesis in beagles. Gut 50: 61–64. 11772968 10.1136/gut.50.1.61 PMC1773071

[11] Mu LN, Cao W, Zhang ZF, Cai L, Jiang QW, et al (2007) Methylenetetrahydrofolate reductase (MTHFR) C677T and A1298C polymorphisms and the risk of primary hepatocellular carcinoma (HCC) in a Chinese population. Cancer Causes Control 18: 665–675. 17503006 10.1007/s10552-007-9012-x PMC4165489

[12] Mu LN, Cao W, Zhang ZF, Yu SZ, Jiang QW, et al (2007) Polymorphisms of 5,10-methylenetetralydrofolate reductase (MTHFR), fruit and vegetable intake, and the risk of stomach cancer. Biomarkers 12: 61–75. 17438654 10.1080/13547500600945101

[13] Chen SY, Chen CJ, Tsai WY, Ahsan H, Liu TY, et al (2000) Associations of plasma aflatoxin B1-albumin adduct level with plasma selenium level and genetic polymorphisms of glutathione S-transferase M1 and T1. Nutr Cancer 38: 179–185. 11525595 10.1207/S15327914NC382_6

[14] Oh SS, Chang SC, Cai L, Cordon-Cardo C, Ding BG, et al (2010) Single nucleotide polymorphisms of 8 inflammation-related genes and their associations with smoking-related cancers. Int J Cancer 127: 2169–2182. 20112337 10.1002/ijc.25214 PMC2932751

[15] Harada S, Zhang S (1993) New strategy for detection of ALDH2 mutant. Alcohol Alcohol Suppl 1A: 11–13. 10.1093/alcalc/28.supplement_1a.11 8141918

[16] Stern LL, Mason JB, Selhub J, Choi SW (2000) Genomic DNA hypomethylation, a characteristic of most cancers, is present in peripheral leukocytes of individuals who are homozygous for the C677T polymorphism in the methylenetetrahydrofolate reductase gene. Cancer Epidemiol Biomarkers Prev 9: 849–853. 10952104

[17] Oh SS, Chang SC, Cai L, Cordon-Cardo C, Ding BG, et al (2010) Single nucleotide polymorphisms of 8 inflammation-related genes and their associations with smoking-related cancers. Int J Cancer 127: 2169–2182. 20112337 10.1002/ijc.25214 PMC2932751

[18] Greenland S (1997) Re: “Estimating relative risk functions in case-control studies using a nonparametric logistic regression”. Am J Epidemiol 146: 883–885. 9384210 10.1093/oxfordjournals.aje.a009208

[19] Robins JM, Greenland S (1992) Identifiability and exchangeability for direct and indirect effects. Epidemiology 3: 143–155. 1576220 10.1097/00001648-199203000-00013

[20] Greenland S (2007) Bayesian perspectives for epidemiological research. II. Regression analysis. Int J Epidemiol 36: 195–202. 17329317 10.1093/ije/dyl289

[21] Efron B, Morris C (1973) Stein's estimation rule and its competitors - an empirical Bayes approach. J Am Stat Assoc 68: 117–130.

[22] Thomas DC, Siemiatycki J, Dewar R, Robins J, Goldberg M, et al (1985) The problem of multiple inference in studies designed to generate hypotheses. Am J Epidemiol 122: 1080–1095. 4061442 10.1093/oxfordjournals.aje.a114189

[23] Greenland S, Robins JM (1991) Empirical-Bayes adjustments for multiple comparisons are sometimes useful. Epidemiology 2: 244–251. 1912039 10.1097/00001648-199107000-00002

[24] Carlin B, Louis TA (2000) Bayes and Empirical-Bayes methods of data analysis. New York: Chapman and Hall.

[25] Sullivan SG, Greenland S (2013) Bayesian regression in SAS software. Int J Epidemiol 42: 308–317. 23230299 10.1093/ije/dys213

[26] Greenland S, Poole C (2013) Living with p values: resurrecting a Bayesian perspective on frequentist statistics. Epidemiology 24: 62–68. 23232611 10.1097/EDE.0b013e3182785741

[27] Greenland S, Poole C (2013) Living with statistics in observational research. Epidemiology 24: 73–78. 23232613 10.1097/EDE.0b013e3182785a49

[28] Garcia-Closas M, Rothman N, Figueroa JD, Prokunina-Olsson L, Han SS, et al (2013) Common genetic polymorphisms modify the effect of smoking on absolute risk of bladder cancer. Cancer Res 73: 2211–2220. 23536561 10.1158/0008-5472.CAN-12-2388 PMC3688270

[29] Rozen R (1997) Genetic predisposition to hyperhomocysteinemia: deficiency of methylenetetrahydrofolate reductase (MTHFR). Thromb Haemost 78: 523–526. 9198208

[30] Bagley PJ, Selhub J (1998) A common mutation in the methylenetetrahydrofolate reductase gene is associated with an accumulation of formylated tetrahydrofolates in red blood cells. Proc Natl Acad Sci U S A 95: 13217–13220. 9789068 10.1073/pnas.95.22.13217 PMC23763

[31] Sohn KJ1, Jang H, Campan M, Weisenberger DJ, Dickhout J, et al (2009) The methylenetetrahydrofolate reductase C677T mutation induces cell-specific changes in genomic DNA methylation and uracil misincorporation: a possible molecular basis for the site-specific cancer risk modification. Int J Cancer 124: 1999–2005. 19123462 10.1002/ijc.24003 PMC2692263

[32] Teng Z, Wang L, Cai S, Yu P, Wang J, et al (2013) The 677C>T (rs1801133) polymorphism in the MTHFR gene contributes to colorectal cancer risk: a meta-analysis based on 71 research studies. PloS one 8: e55332. 23437053 10.1371/journal.pone.0055332 PMC3577825

[33] Yu L, Chen J (2012) Association of MTHFR Ala222Val (rs1801133) polymorphism and breast cancer susceptibility: An update meta-analysis based on 51 research studies. Diagnostic pathology 7: 171. 23217001 10.1186/1746-1596-7-171 PMC3536596

[34] You W, Li Z, Jing C, Qian-Wei X, Yu-Ping Z, et al (2013) MTHFR C677T and A1298C polymorphisms were associated with bladder cancer risk and disease progression: a meta-analysis. DNA Cell Biol 32: 260–267. 23578207 10.1089/dna.2012.1931

[35] Yu L, Chang K, Han J, Deng S, Chen M (2013) Association between Methylenetetrahydrofolate reductase C677T polymorphism and susceptibility to cervical cancer: a meta-analysis. PloS one 8: e55835. 23431363 10.1371/journal.pone.0055835 PMC3576378

[36] Liu ZB, Wang LP, Shu J, Jin C, Lou ZX (2013) Methylenetetrahydrofolate reductase 677TT genotype might be associated with an increased lung cancer risk in Asians. Gene 515: 214–219. 23237779 10.1016/j.gene.2012.11.036

[37] Ibiebele TI, Hughes MC, Pandeya N, Zhao Z, Montgomery G, et al (2011) High intake of folate from food sources is associated with reduced risk of esophageal cancer in an Australian population. J Nutr 141: 274–283. 21178085 10.3945/jn.110.131235 PMC3021447

[38] Marchal C, Redondo M, Reyes-Engel A, Perea-Milla E, Gaitan MJ, et al (2008) Association between polymorphisms of folate-metabolizing enzymes and risk of prostate cancer. Eur J Surg Oncol 34: 805–810. 17967524 10.1016/j.ejso.2007.09.008

[39] Moore LE, Hung R, Karami S, Boffetta P, Berndt S, et al (2008) Folate metabolism genes, vegetable intake and renal cancer risk in central Europe. Int J Cancer 122: 1710–1715. 18098291 10.1002/ijc.23318

[40] Moore LE, Malats N, Rothman N, Real FX, Kogevinas M, et al (2007) Polymorphisms in one-carbon metabolism and trans-sulfuration pathway genes and susceptibility to bladder cancer. Int J Cancer 120: 2452–2458. 17311259 10.1002/ijc.22565

[41] Ott N, Geddert H, Sarbia M (2008) Polymorphisms in methionine synthase (A2756G) and cystathionine beta-synthase (844ins68) and susceptibility to carcinomas of the upper gastrointestinal tract. J Cancer Res Clin Oncol 134: 405–410. 17726616 10.1007/s00432-007-0301-2 PMC12161651

[42] Suzuki T, Matsuo K, Hasegawa Y, Hiraki A, Wakai K, et al (2007) One-carbon metabolism-related gene polymorphisms and risk of head and neck squamous cell carcinoma: case-control study. Cancer Sci 98: 1439–1446. 17596206 10.1111/j.1349-7006.2007.00533.x PMC11158578

[43] Suzuki T, Matsuo K, Hiraki A, Saito T, Sato S, et al (2007) Impact of one-carbon metabolism-related gene polymorphisms on risk of lung cancer in Japan: a case control study. Carcinogenesis 28: 1718–1725. 17468511 10.1093/carcin/bgm104

[44] Zhang FF, Terry MB, Hou L, Chen J, Lissowska J, et al (2007) Genetic polymorphisms in folate metabolism and the risk of stomach cancer. Cancer Epidemiol Biomarkers Prev 16: 115–121. 17220339 10.1158/1055-9965.EPI-06-0513

[45] Zhong S, Xu J, Li W, Chen Z, Ma T, et al (2013) Methionine synthase A2756G polymorphism and breast cancer risk: an up-to-date meta-analysis. Gene 527: 510–515. 23845785 10.1016/j.gene.2013.06.054

[46] Ding W, Zhou DL, Jiang X, Lu LS (2013) Methionine synthase A2756G polymorphism and risk of colorectal adenoma and cancer: evidence based on 27 studies. PloS one 8: e60508. 23593229 10.1371/journal.pone.0060508 PMC3621882

[47] Olteanu H, Munson T, Banerjee R (2002) Differences in the efficiency of reductive activation of methionine synthase and exogenous electron acceptors between the common polymorphic variants of human methionine synthase reductase. Biochemistry 41: 13378–13385. 12416982 10.1021/bi020536s

[48] Kwak SY, Kim UK, Cho HJ, Lee HK, Kim HJ, et al (2008) Methylenetetrahydrofolate reductase (MTHFR) and methionine synthase reductase (MTRR) gene polymorphisms as risk factors for hepatocellular carcinoma in a Korean population. Anticancer Res 28: 2807–2811. 19035314

[49] Han D, Shen C, Meng X, Bai J, Chen F, et al (2012) Methionine synthase reductase A66G polymorphism contributes to tumor susceptibility: evidence from 35 case-control studies. Mol Biol Rep 39: 805–816. 21547363 10.1007/s11033-011-0802-6

[50] Jokic M, Brcic-Kostic K, Stefulj J, Catela Ivkovic T, Bozo L, et al (2011) Association of MTHFR, MTR, MTRR, RFC1, and DHFR gene polymorphisms with susceptibility to sporadic colon cancer. DNA Cell Biol 30: 771–776. 21438757 10.1089/dna.2010.1189

[51] Pardini B, Kumar R, Naccarati A, Prasad RB, Forsti A, et al (2011) MTHFR and MTRR genotype and haplotype analysis and colorectal cancer susceptibility in a case-control study from the Czech Republic. Mutation research 721: 74–80. 21211571 10.1016/j.mrgentox.2010.12.008

[52] Stolzenberg-Solomon RZ, Qiao YL, Abnet CC, Ratnasinghe DL, Dawsey SM, et al (2003) Esophageal and gastric cardia cancer risk and folate- and vitamin B(12)-related polymorphisms in Linxian, China. Cancer Epidemiol Biomarkers Prev 12: 1222–1226. 14652285

[53] Yoo JY, Kim SY, Hwang JA, Hong SH, Shin A, et al (2012) Association Study between Folate Pathway Gene Single Nucleotide Polymorphisms and Gastric Cancer in Koreans. Genomics Inform 10: 184–193. 23166529 10.5808/GI.2012.10.3.184 PMC3492654

[54] Hazra A, Fuchs CS, Kawasaki T, Kirkner GJ, Hunter DJ, et al (2010) Germline polymorphisms in the one-carbon metabolism pathway and DNA methylation in colorectal cancer. Cancer Causes Control 21: 331–345. 19936946 10.1007/s10552-009-9464-2 PMC3570978

[55] Lissowska J, Gaudet MM, Brinton LA, Chanock SJ, Peplonska B, et al (2007) Genetic polymorphisms in the one-carbon metabolism pathway and breast cancer risk: a population-based case-control study and meta-analyses. Int J Cancer 120: 2696–2703. 17311260 10.1002/ijc.22604

[56] Shen M, Rothman N, Berndt SI, He X, Yeager M, et al (2005) Polymorphisms in folate metabolic genes and lung cancer risk in Xuan Wei, China. Lung Cancer 49: 299–309. 15922487 10.1016/j.lungcan.2005.04.002

[57] Matsuo K, Ito H, Wakai K, Hirose K, Saito T, et al (2005) One-carbon metabolism related gene polymorphisms interact with alcohol drinking to influence the risk of colorectal cancer in Japan. Carcinogenesis 26: 2164–2171. 16051637 10.1093/carcin/bgi196

[58] Crabb DW, Edenberg HJ, Bosron WF, Li TK (1989) Genotypes for aldehyde dehydrogenase deficiency and alcohol sensitivity. The inactive ALDH2(2) allele is dominant. J Clin Invest 83: 314–316. 2562960 10.1172/JCI113875 PMC303676

[59] Cadoni G, Boccia S, Petrelli L, Di Giannantonio P, Arzani D, et al (2012) A review of genetic epidemiology of head and neck cancer related to polymorphisms in metabolic genes, cell cycle control and alcohol metabolism. Acta Otorhinolaryngol Ital 32: 1–11. 22500060 PMC3324962

[60] Yang SJ, Yokoyama A, Yokoyama T, Huang YC, Wu SY, et al (2010) Relationship between genetic polymorphisms of ALDH2 and ADH1B and esophageal cancer risk: a meta-analysis. World J Gastroenterol 16: 4210–4220. 20806441 10.3748/wjg.v16.i33.4210 PMC2932928

[61] Chou WY, Stewart MJ, Carr LG, Zheng D, Stewart TR, et al (1999) An A/G polymorphism in the promoter of mitochondrial aldehyde dehydrogenase (ALDH2): effects of the sequence variant on transcription factor binding and promoter strength. Alcohol Clin Exp Res 23: 963–968. 10397279

[62] Bye H, Prescott NJ, Matejcic M, Rose E, Lewis CM, et al (2011) Population-specific genetic associations with oesophageal squamous cell carcinoma in South Africa. Carcinogenesis 32: 1855–1861. 21926110 10.1093/carcin/bgr211 PMC3220606

[63] Ma WJ, Lv GD, Zheng ST, Huang CG, Liu Q, et al (2010) DNA polymorphism and risk of esophageal squamous cell carcinoma in a population of North Xinjiang, China. World J Gastroenterol 16: 641–647. 20128036 10.3748/wjg.v16.i5.641 PMC2816280

[64] Duell EJ, Sala N, Travier N, Munoz X, Boutron-Ruault MC, et al (2012) Genetic variation in alcohol dehydrogenase (ADH1A, ADH1B, ADH1C, ADH7) and aldehyde dehydrogenase (ALDH2), alcohol consumption and gastric cancer risk in the European Prospective Investigation into Cancer and Nutrition (EPIC) cohort. Carcinogenesis 33: 361–367. 22144473 10.1093/carcin/bgr285

[65] Zhang FF, Hou L, Terry MB, Lissowska J, Morabia A, et al (2007) Genetic polymorphisms in alcohol metabolism, alcohol intake and the risk of stomach cancer in Warsaw, Poland. Int J Cancer 121: 2060–2064. 17631643 10.1002/ijc.22973

[66] Yang H, Zhou Y, Zhou Z, Liu J, Yuan X, et al (2009) A novel polymorphism rs1329149 of CYP2E1 and a known polymorphism rs671 of ALDH2 of alcohol metabolizing enzymes are associated with colorectal cancer in a southwestern Chinese population. Cancer Epidemiol Biomarkers Prev 18: 2522–2527. 19706845 10.1158/1055-9965.EPI-09-0398

[67] Canova C, Richiardi L, Merletti F, Pentenero M, Gervasio C, et al (2010) Alcohol, tobacco and genetic susceptibility in relation to cancers of the upper aerodigestive tract in northern Italy. Tumori 96: 1–10. 20437850 10.1177/030089161009600101

[68] Chung CS, Lee YC, Liou JM, Wang CP, Ko JY, et al (2012) Tag single nucleotide polymorphisms of alcohol-metabolizing enzymes modify the risk of upper aerodigestive tract cancers: HapMap database analysis. Dis Esophagus 27: 493–503. 23088731 10.1111/j.1442-2050.2012.01437.x

[69] Hashibe M, Boffetta P, Zaridze D, Shangina O, Szeszenia-Dabrowska N, et al (2006) Evidence for an important role of alcohol- and aldehyde-metabolizing genes in cancers of the upper aerodigestive tract. Cancer Epidemiol Biomarkers Prev 15: 696–703. 16614111 10.1158/1055-9965.EPI-05-0710

[70] Greenland S (2003) Quantifying biases in causal models: classical confounding vs collider-stratification bias. Epidemiology 14: 300–306. 12859030

[71] Fredriksen A, Meyer K, Ueland PM, Vollset SE, Grotmol T, et al (2007) Large-scale population-based metabolic phenotyping of thirteen genetic polymorphisms related to one-carbon metabolism. Human mutation 28: 856–865. 17436311 10.1002/humu.20522

