# Supplementary material for: Single Nucleotide Polymorphisms of One-Carbon Metabolism and Cancers of the Esophagus, Stomach, and Liver in a Chinese Population
Source: PLoS One. 2014 Oct 22;9(10):e109235. doi: 10.1371/journal.pone.0109235 (PMC4206280; doi:10.1371/journal.pone.0109235)
Supplement: Table S2 — Results of the associations between SNPs of MTHFR, MTR, MTRR, DNMT1, and ALDH2 genes and cancers of esophagus, stomach, and liver, stratified on plasma vitamin B12 levels. (DOC) [file pone.0109235.s002.doc]

**Table S2. Associations between SNPs of MTHFR, MTR, MTRR, DNMT1, and ALDH2 genes and cancers of the esophagus, stomach, and liver, stratified on plasma vitamin B12** levels†

| **Plasma folate levels** | **Esophageal cancer** | | | | **Stomach cancer** | | | | | **Liver cancer** | | | | |
| --- | --- | --- | --- | --- | --- | --- | --- | --- | --- | --- | --- | --- | --- | --- |
|  | **≤228.88 pmol/l** | | **> 228.88 pmol/l** | | **≤228.88 pmol/l** | | **> 228.88 pmol/l** | | | **≤228.88 pmol/l** | | | **> 228.88 pmol/l** | |
|  | **SBOR***  **(95% posterior limits)** | **One- sided *P^‡^*** | **SBOR***  **(95% posterior limits)** | **One- sided *P^‡^*** | **SBOR***  **(95% posterior limits)** | **One- sided *P^‡^*** | **SBOR***  **(95% posterior limits)** | | **One- sided *P^‡^*** | **SBOR***  **(95% posterior limits)** | | **One- sided *P^‡^*** | **SBOR***  **(95% posterior limits)** | **One- sided *P^‡^*** |
| **MTHFR** |  |  |  |  |  |  |  | |  |  | |  |  |  |
| rs1801133 | 0.96 (0.55, 1.70) | 0.45 | 1.31 (0.82, 2.07) | 0.13 | 1.39 (0.80, 2.42) | 0.12 | 1.69 (1.03, 2.77) | | 0.018 | 0.93 (0.48, 1.78) | | 0.41 | 1.59 (0.99, 2.57) | 0.029 |
|  | *P* for heterogeneity = 0.44 | | | | *P* for heterogeneity = 0.69 | | | | | *P* for heterogeneity = 0.26 | | | | |
| **MTR** |  |  |  |  |  |  |  | |  |  | |  |  |  |
| rs1805087 | 0.84 (0.44, 1.58) | 0.29 | 1.03 (0.60, 1.75) | 0.46 | 1.01 (0.56, 1.83) | 0.49 | | 1.26 (0.74, 2.15) | 0.20 | 0.82 (0.41, 1.65) | 0.29 | | 1.06 (0.62, 1.80) | 0.42 |
|  | *P* for heterogeneity = 0.61 | | | | *P* for heterogeneity = 0.61 | | | | | *P* for heterogeneity = 0.55 | | | | |
| **MTRR** |  |  |  |  |  |  |  | |  |  | |  |  |  |
| rs1532268 | 2.15 (0.94, 4.95) | 0.035 | 0.98 (0.63, 1.53) | 0.48 | 1.29 (0.54, 3.09) | 0.28 | 0.82 (0.36, 1.86) | | 0.32 | 0.95 (0.36, 2.48) | | 0.46 | 0.99 (0.45, 2.19) | 0.49 |
|  | *P* for heterogeneity = 0.11 | | | | *P* for heterogeneity = 0.45 | | | | | *P* for heterogeneity = 0.89 | | | | |
| rs1801394 | 0.90 (0.51, 1.57) | 0.35 | 0.98 (0.46, 2.11) | 0.47 | 0.71 (0.41, 1.21) | 0.10 | 0.81 (0.51, 1.30) | | 0.19 | 1.10 (0.44, 2.77) | | 0.42 | 1.12 (0.60, 2.12) | 0.36 |
|  | *P* for heterogeneity = 0.77 | | | | *P* for heterogeneity = 0.65 | | | | | *P* for heterogeneity = 0.85 | | | | |
| **DNMT1** |  |  |  |  |  |  |  | |  |  | |  |  |  |
| rs2228612 | 0.71 (0.37, 1.35) | 0.15 | 0.81 (0.49, 1.34) | 0.21 | 1.08 (0.56, 2.10) | 0.41 | 1.25 (0.68, 2.28) | | 0.24 | 1.64 (0.76, 3.54) | | 0.10 | 1.25 (0.72, 2.18) | 0.22 |
|  | *P* for heterogeneity = 0.63 | | | | *P* for heterogeneity = 0.79 | | | | | *P* for heterogeneity = 0.35 | | | | |
| **ALDH2** |  |  |  |  |  |  |  | |  |  | |  |  |  |
| rs671 | 1.80 (0.83, 3.90) | 0.068 | 1.11 (0.58, 2.13) | 0.38 | 1.15 (0.49, 2.66) | 0.38 | 0.89 (0.44, 1.77) | | 0.37 | 1.14 (0.48, 2.74) | | 0.38 | 0.83 (0.40, 1.68) | 0.30 |
|  | *P* for heterogeneity = 0.24 | | | | *P* for heterogeneity = 0.65 | | | | | *P* for heterogeneity = 0.62 | | | | |
| rs2238151 | 0.63 (0.28, 1.39) | 0.12 | 0.91 (0.45, 1.83) | 0.39 | 0.80 (0.39, 1.66) | 0.28 | 1.51 (0.77, 2.95) | | 0.12 | 1.00 (0.38, 2.66) | | 0.50 | 1.16 (0.45, 2.99) | 0.38 |
|  | *P* for heterogeneity = 0.39 | | | | *P* for heterogeneity = 0.22 | | | | | *P* for heterogeneity = 1.00 | | | | |
| rs886205 | 0.71 (0.37, 1.37) | 0.15 | 1.02 (0.58, 1.78) | 0.47 | 0.90 (0.48, 1.68) | 0.37 | 1.87 (1.09, 3.20) | | 0.011 | 1.36 (0.69, 2.70) | | 0.19 | 1.42 (0.83, 2.43) | 0.10 |
|  | *P* for heterogeneity = 0.38 | | | | *P* for heterogeneity = 0.097 | | | | | *P* for heterogeneity = 0.90 | | | | |

*: Semi-Bayes odds ratio (SBOR) adjusted for age (5-year categories and deviation from stratum mean), sex, residency (city, rural), alcohol drinking frequency, smoking pack-years, BMI, education, H. *pylori* infection (in stomach cancer analyses), HBsAg (in liver cancer analyses ), and plasma AFB1 levels (in liver cancer analyses).

†: SNP-cancer associations were under dominant genetic models, except for rs1532268 with three cancers, rs1801394 with liver cancer, rs2228612 with stomach and liver cancers, rs671 with three cancers, and rs2238151 with liver cancer, which are under recessive genetic models.

‡: One-sided semi-Bayes *P*-values; the posterior probability that the point estimate is on the wrong side of the null.
