# Supplementary material for: Single Nucleotide Polymorphisms of One-Carbon Metabolism and Cancers of the Esophagus, Stomach, and Liver in a Chinese Population
Source: PLoS One. 2014 Oct 22;9(10):e109235. doi: 10.1371/journal.pone.0109235 (PMC4206280; doi:10.1371/journal.pone.0109235)
Supplement: Table S6 — Results of the associations between SNPs of MTHFR, MTR, MTRR, DNMT1, and ALDH2 genes and stomach cancer, stratified on H. Pylori infection status, and on liver cancer, stratified on HBsAg status and plasma aflatoxin B1 albumin adduct levels. (DOC) [file pone.0109235.s006.doc]

**Table S6. Associations between SNPs of MTHFR, MTR, MTRR, DNMT1, and ALDH2 genes and cancers of the stomach, stratified on H. *pylori* infection status, and liver, stratified on HBsAg status and plasma AFB1 albumin adduct levels**†

| **Plasma folate levels** | **Stomach cancer** | | | | **Liver cancer** | | | | **Liver cancer** | | | |
| --- | --- | --- | --- | --- | --- | --- | --- | --- | --- | --- | --- | --- |
|  | **H. *pylori* CagA̶** | | **H. *pylori* CagA+** | | **HBsAg ̶** | | **HBsAg +** | | **AFB1 Lower** | | **AFB1 Higher** | |
|  | **SBOR***  **(95% posterior limits)** | **One- sided *P^‡^*** | **SBOR***  **(95% posterior limits)** | **One- sided *P^‡^*** | **SBOR***  **(95% posterior limits)** | **One- sided *P^‡^*** | **SBOR***  **(95% posterior limits)** | **One- sided *P^‡^*** | **SBOR***  **(95% posterior limits)** | **One- sided *P^‡^*** | **SBOR***  **(95% posterior limits)** | **One- sided *P^‡^*** |
| **MTHFR** |  |  |  |  |  |  |  |  |  |  |  |  |
| rs1801133 | 1.55 (0.98, 2.45) | 0.032 | 1.64 (0.90, 2.98) | 0.053 | 1.41 (0.83, 2.39) | 0.10 | 1.35 (0.79, 2.30) | 0.13 | 1.33 (0.75, 2.35) | 0.16 | 1.29 (0.75, 2.22) | 0.18 |
|  | *P* for heterogeneity = 0.68 | | | | *P* for heterogeneity = 0.91 | | | | *P* for heterogeneity = 0.91 | | | |
| **MTR** |  |  |  |  |  |  |  |  |  |  |  |  |
| rs1805087 | 1.11 (0.68, 1.82) | 0.33 | 1.20 (0.62, 2.31) | 0.29 | 1.18 (0.66, 2.12) | 0.29 | 0.78 (0.45, 1.36) | 0.19 | 1.03 (0.57, 1.86) | 0.46 | 0.96 (0.53, 1.77) | 0.45 |
|  | *P* for heterogeneity = 0.78 | | | | *P* for heterogeneity = 0.32 | | | | *P* for heterogeneity = 0.88 | | | |
| **MTRR** |  |  |  |  |  |  |  |  |  |  |  |  |
| rs1532268 | 0.98 (0.43, 2.20) | 0.48 | 1.08 (0.45, 2.60) | 0.43 | 1.12 (0.49, 2.52) | 0.40 | 0.97 (0.38, 2.45) | 0.47 | 1.46 (0.59, 3.58) | 0.21 | 0.71 (0.29, 1.70) | 0.22 |
|  | *P* for heterogeneity = 0.85 | | | | *P* for heterogeneity = 0.87 | | | | *P* for heterogeneity = 0.26 | | | |
| rs1801394 | 0.66 (0.42, 1.02) | 0.030 | 1.02 (0.58, 1.81) | 0.47 | 1.23 (0.62, 2.45) | 0.28 | 1.35 (0.60, 3.03) | 0.23 | 1.14 (0.52, 2.51) | 0.37 | 1.42 (0.69, 2.94) | 0.17 |
|  | *P* for heterogeneity = 0.27 | | | | *P* for heterogeneity = 0.69 | | | | *P* for heterogeneity = 0.76 | | | |
| **DNMT1** |  |  |  |  |  |  |  |  |  |  |  |  |
| rs2228612 | 1.10 (0.61, 1.98) | 0.37 | 1.28 (0.64, 2.54) | 0.24 | 1.74 (0.94, 3.20) | 0.039 | 1.07 (0.58, 1.96) | 0.42 | 1.22 (0.64, 2.34) | 0.27 | 1.31 (0.70, 2.42) | 0.20 |
|  | *P* for heterogeneity = 0.69 | | | | *P* for heterogeneity = 0.26 | | | | *P* for heterogeneity = 0.91 | | | |
| **ALDH2** |  |  |  |  |  |  |  |  |  |  |  |  |
| rs671 | 0.81 (0.39, 1.69) | 0.28 | 1.22 (0.56, 2.63) | 0.31 | 0.61 (0.27, 1.40) | 0.12 | 1.46 (0.68, 3.11) | 0.17 | 0.86 (0.40, 1.85) | 0.35 | 1.23 (0.53, 2.86) | 0.31 |
|  | *P* for heterogeneity = 0.45 | | | | *P* for heterogeneity = 0.13 | | | | *P* for heterogeneity = 0.53 | | | |
| rs2238151 | 1.08 (0.57, 2.05) | 0.41 | 1.17 (0.55, 2.50) | 0.34 | 1.21 (0.47, 3.15) | 0.35 | 1.00 (0.38, 2.66) | 0.50 | 1.00 (0.38, 2.66) | 0.50 | 1.19 (0.46, 3.07) | 0.36 |
|  | *P* for heterogeneity = 0.81 | | | | *P* for heterogeneity = 1.00 | | | | *P* for heterogeneity = 1.00 | | | |
| rs886205 | 1.59 (0.95, 2.64) | 0.038 | 1.03 (0.55, 1.95) | 0.46 | 1.43 (0.83, 2.46) | 0.10 | 1.14 (0.62, 2.11) | 0.34 | 1.20 (0.64, 2.23) | 0.28 | 1.35 (0.74, 2.47) | 0.16 |
|  | *P* for heterogeneity = 0.38 | | | | *P* for heterogeneity = 0.65 | | | | *P* for heterogeneity = 0.80 | | | |

*: Semi-Bayes odds ratio (SBOR) adjusted for age (5-year categories and deviation from stratum mean), sex, residency (city, rural), alcohol drinking frequency, smoking pack-years, BMI, education, HBsAg (in stratified analyses on plasma AFB1 levels), and plasma AFB1levels (in stratified analyses on HBsAg status).

†: SNP-cancer associations were under dominant genetic models, except for rs1532268 with stomach and liver cancers, rs1801394 with liver cancer, rs2228612 with stomach and liver cancers, rs671 with stomach and liver cancers, and rs2238151 with liver cancer, which are under recessive genetic models.

‡: One-sided semi-Bayes *P*-values; the posterior probability that the point estimate is on the wrong side of the null.
